# When clean eating isn’t as faultless: the dangerous obsession with healthy eating and the relationship between Orthorexia nervosa and eating disorders in Mexican University students

**DOI:** 10.1186/s40337-020-00331-2

**Published:** 2020-10-26

**Authors:** Alicia Parra Carriedo, Antonio Tena-Suck, Miriam Wendolyn Barajas-Márquez, Gladys María Bilbao y Morcelle, Mary Carmen Díaz Gutiérrez, Isabel Flores Galicia, Alejandra Ruiz-Shuayre

**Affiliations:** 1 Universidad Iberoamericana CDMX, México, Mexico; 2 Universidad Nacional Autónoma de México, Mexico, Mexico

**Keywords:** ORTO-14 MX, ortorexia nervosa, trastornos de la conducta alimentaria, población mexicana, instrumentos de tamizaje, ORTO-14MX, Orthorexia nervosa, Eating disorders, Mexican population, Screening instrument

## Abstract

**Background:**

Orthorexia Nervosa is an eating disorder that has been scarcely studied in characteristics, causes and symptoms, as well as in consequences and the relationship with other eating disorders. The present study had as its main objective the analysis of said relationship and inquisition of the possibility of predicting the development of an eating disorder from the presence of orthorexia nervosa. Also, it analyzed the differences by sex in Mexican university students.

**Methods:**

The sample consisted of 911 university students (65.4% women and 34.6% men), between an age range of 18 to 28 years old (M = 21 and SD = 1.9). Two questionnaires were responded: the ORTO14MX, a version of the ORTO-15 instrument previously validated in Mexican students, and the EDE-Q in its short version. Besides, sociodemographic data of interest was obtained and used for this study.

**Results:**

The Pearson’s correlation analysis demonstrated statistically significant relations, from mild to moderate, between the factors that make up both scales, while the linear regression analysis demonstrated that 40% of the variance is explained with the factors from the ORTO-14MX scale in the overall sample for the study. Additionally, statistically significant differences were found between men and women regarding all the subscales conforming the instruments that measured orthorexia and eating disorders.

**Conclusion:**

These results show a moderate predictive degree, that as promising as it is, isn’t conclusive. Likewise, it was confirmed that women are still more vulnerable to develop orthorexia or another eating disorder. It can be concluded that there’s a vast necessity for more studies measuring the relationship between orthorexia nervosa and eating disorders, in Latinamerican samples with diverse characteristics in sociocultural backgrounds, and clinical samples.

## Resumen

**Introducción:**

La ortorexia nervosa es un trastorno de la conducta alimentaria que ha sido poco estudiado, tanto en sus características, causas y síntomas, como en sus consecuencias y en la relación con otros trastornos de la conducta alimentaria. El presente estudio tuvo como objetivo analizar dicha relación e indagar si es posible predecir la aparición de un trastorno de la conducta alimentaria a partir de la presencia de ortorexia nervosa, además de analizar diferencias por sexo en estudiantes universitarios mexicanos.

**Método:**

Participaron en el estudio 911 estudiantes universitarios (65.4% fueron mujeres y el 34.6% hombres), en un rango de edades entre 18 y 28 años (M = 21 y DE = 1.9) que respondieron dos cuestionarios que son: ORTO-14MX, una versión del instrumento ORTO-15 previamente validada en estudiantes mexicanos y el EDE-Q en su versión corta, además de proporcionar algunos datos sociodemográficos de interés para este estudio.

**Resultados:**

El análisis de correlación de Pearson reveló relaciones estadísticas significativas de leves a moderadas entre los factores que componen ambas escalas, mientras que el análisis de regresión lineal mostró un que un 40% de la varianza es explicada a partir de los factores que componen la escala ORTO-14MX en la muestra de estudio. Adicionalmente se encontraron diferencias estadísticamente significativas entre hombres y mujeres con respecto a todas las subescalas de los instrumentos que midieron ortorexia y trastornos de la conducta alimentaria.

**Conclusiones:**

Estos resultados mostraron un grado de predicción moderada que sí bien es prometedora, no resulta totalmente concluyente. Asimismo, se confirmó que las mujeres siguen siendo más vulnerables a padecer ortorexia o un TCA. Se concluye la necesidad de continuar estudiando la relación entre la ortorexia y los TCAs, en muestras latinoamericanas con otras características socio demográficas y en población clínica.

## Plain English summary

The present article explores various aspects regarding orthorexia and eating disorders in Mexican university students. The relationship between both is explored, as well as social and cultural variables that could describe their appearance and development. The ORTO-14MX scale and EDE-Q in its short version were used to grade diverse proneness and traits to both phenomena, and to find out if there was a connection between them, and if ORTO-14MX could predict EDE-Q. Also, there were differences found from men and women, with women being more susceptible to develop orthorexia, or any other eating disorder. In conclusion, psychosocial factors, as well as age and culture, influence eating habits. More studies are definitely needed to understand the experience of having disordered eating habits, particularly for Latinamerican samples.

## Background

In recent years, the term “Orthorexia Nervosa” (ON) has shown an increase in literature. The word “orthorexia” comes from the Greek “orthos” (“correct”), and “orexia” (“appetite”, “nutrition”), meaning overall “correct nutrition”. The term was first described by Steven Bratman in 1997 as “having a pathological obsession in pursuit of a healthy diet” [1]. This concept indicates a possible new eating disorder (ED). Its main symptom is an obsessive and pathological approach to eating what is perceived as “healthy” [2].

The inclination to eat healthily is perceived as a desirable behavior in general. However, when it acquires obsessive traits and prevents the person from leading a healthy life, it is a risky behavior, and can even lead to malnutrition [3, 4]. This is why these orthorexic behaviors can be confused with anorexic behaviors (for example), or even be ignored, encouraged, or praised as a way of taking care of themselves and their body. Thus, the relation between ON and ED is complex and unequivocal; and still far from being completely understood.

Some of the shared traits between ON and ED’s that have been previously reported are: a cognitive fixation on nutrition, perfectionism, high anxiety levels, the need to keep everything under control, feeling guilty if they had transgressions with their diet, self-discipline actions in direct proportion to adherence to the diet, cognitive rigidity, and denial of the functional deficiencies associated to the disorder [5]. However, ON does not include the primary symptoms of anorexia nervosa (AN) and bulimia nervosa (BN), which are fear of gaining weight, an excessive obsession with being slim, and a distorted perception of the body [3]. Varga et al., point out that even if the difference between ON and EDs resides effectively in the final motivation (weight loss for EDs, and “feeling healthy” for ON), the social and psychological consequences are similar [6].

It has been proposed that ON could precede the development of an ED, or could as well present itself as the evolution of an ED on remission and rehabilitation, allowing an individual with a previously diagnosed ED, to feel accepted by society again. Hence, this would serve as an approved way to maintain control over the body and food [7]. Barthels et al. [8] believe that eating behaviors consistent with ON’s symptomatology can be associated with a certain level of recovery eating disorders and what he considers can be a shift to “less grave” ED behaviors. This suggests that ON could be a way for people with AN to recover [9]. In a study led by Segura-Garcia et al., it was observed that 28% of patients with AN and BN showed tendencies of ON. Additionally, they observed that the proportion of patients with ON tendencies increased up to a 53% by the termination of treatment for an ED [7]. Another study carried out demonstrated that 67% of the professionals in charge of treating patients with ED observed ON behaviors in their clinical practice, and 69% considered that ON as a disorder deserves, and requires, more attention [9].

Despite its similarities with AN, ON is not yet recognized as an eating disorder in the DSM-5 (Diagnostic and Statistical Manual of Mental Disorders), nor in the ICD-10 (International Classification of Diseases) [2, 10]. Therefore, there is no consensus to date, neither in the diagnostic criteria nor in the evaluation procedures for ON.

There are various attempts by a couple of authors to try to establish the diagnostic criteria of ON. There authors are: Setnick et al. [11], Moroze et al. [3], Barthels et al. [8], and Dunn and Bratman [12], amongst others. Out of the proposed criteria, two main characteristics must be included:

- An obsessive approach to food and eating practices, to promote optimal well-being through a diet considered as healthy (but is inflexible and shows persistent and recurring concern over food).
- Significant clinical deterioration, which may include medical, nutritional, and/or psychological complications, high levels of distress, and/or decline in important areas of social functioning [2].

As for the evaluation procedures, ON has been measured with the Bratman’s Orthorexia Test (BOT) [1] and the ORTO-15 [13], the latter based on the former. Both instruments have been translated into several languages and applied in clinical samples and according to personal convenience. The limited existing literature on the prevalence of ON has been primarily written based on the ORTO-15, reporting a prevalence ranging from 6.9% [14] up to 57.6% [4]. Currently, there is little to no research with representative samples studying the prevalence of ON in the general mexican population. Besides, most studies have been conducted with high-risk groups [6], mainly university students [9, 15, 16].

The teen and college-aged population is a particularly vulnerable group from a nutritional point of view, since they’re individuals who are starting to take responsibility over their own eating habits. Because of this, this time becomes a critical period in the consolidation of habits and eating behaviors, as well as the development of their body image [17, 18]. If it is considered that most of the behaviors established during the teenage years persist throughout development, this period represents a valuable opportunity to evaluate predictors and risk factors for the development of an ED [19]. It is because of this that most studies that have taken place, on ON and on the validation of the tools used for its detection, have been on college students [20]. Within the group of college students there have even been subgroups described, which would be considered even more at risk, such as Nutrition, Medicine and Sport Sciences students [21–25]. They present a higher risk because these students have more awareness and information on healthy eating [24, 26–28]. Other groups with higher risks would be people who give great importance to body image, such as actors, dancers, and athletes [29–31], and people who previously had an ED [32].

Previous studies that have analyzed the appearance and symptoms of ON have mainly focused on the eating behaviors, evaluating ON’s tendency in groups of people who follow a special diet. In a study led by Plichta et al., it was observed that the risk of showing a tendency toward ON and symptoms of an ED was less in students who did not follow a diet previously or at the time of the study (OR: 0.34 and 0.26 respectively), in comparison to students who had followed a diet previously or at the time of the study. This suggests that the use of diets in the past or present must be considered as well as an important predictor of the presence of tendencies toward ON [33].

In another study also conducted by Plichta et al., where 1120 university students from Poland were evaluated (546 students from health-related careers and 573 students from other areas), it was observed that 46.7% of the population had a score in the ORTO-15 ranging between 35 and 40, regardless of their degree (indicative of ON). However, within this percentage range, there were more students from health-related degrees than students from other areas [34].

In another study carried out by Malmborg et al., the incidence of ON was evaluated in university students in Sweden. These students belonged to health-related careers (exercise physiology, nutrition, and health), and students from business-related areas (finance, marketing, and accounting). The authors reported that 76.6% of the full population presented scores lower than 40 (< 40) in the ORTO-15, an indicative of ON. Consistently with other studies and data in literature, higher prevalence was observed in students from health-related careers than in students from business-related areas (84.5% vs. 65.5%) [25].

It is important to emphasize that there are rather few studies that have tested for ON in the general population [6]; therefore, more studies are required to better comprehend what the possible implications of ON are for them.

Eating behaviors are profoundly molded by the context in which the person lives, including the cultural, social, and ecological environments [35]. The interaction between the sociocultural context and ON has been scarcely studied, too. Most studies done on ON have been tried on European, Turkish, or American populations [36]. There are few studies which have been developed with Brazilian or Latinamerican populations [37] Partly, the observed differences in prevalence in studies done in different countries could be explained by the relation between the sociocultural factors and the eating habits of each country [38, 39].

It would be of great interest to understand the differences in the cultural backdrop that exist in terms of what the students consider to be a healthy lifestyle and how to achieve it. It was observed in a study led by Gramaglia et al., in which a transcultural comparison was made between university students in Spain, Italy and Poland, that a greater prevalence of ON existed in the Polish sample, in contrast to the Spanish and Italian ones. One of the proposed explanations for the findings is that Polish students believe that a healthy diet is the best way to improve their health, consequently having good consumption practices and control over the composition of their products. In contrast, Spanish and Italian people have a wine and food culture based on a Mediterranean diet, and value more the social aspects of having a meal [40].

Thus, the objective of this study in its first stance, was to analyze the existing relationship between the composing factors of the ORTO-14MX and the factors from the Eating Disorders Questionnaire (EDE-Q) in its short version, to later find out if these factors that indicate orthorexia nervosa (ON) accurately predict eating disorders in university students from Mexico City. Additionally, we tested for differences in these same variables between men and women, according to these same variables.

## Method

### Type of study

This non-experimental cross-sectional study was conducted during Summer 2018. Specifically, the dates in which the study was carried out were May 15 to July 15.

### Participants

The sample consisted of 911 university students (65.4% women and 34.6% men), between an age range of 18 to 28 years old (M = 21 and SD = 1.9). These students belonged to different majors at bachelor and specialty levels. Out of these, 27.8% studied Nutrition, 20.5% Social Sciences, 16.9% Arts and Humanities, 15.6% Engineering, 9.9% Economic-Administrative careers, and 9.4% Health Sciences. These students were taking Summer classes from May to July in 2018.

The average BMI in women was 21.8 (SD = 3.08), while in men the average was 23.4 (SD = 3.41); this can be seen in Table 1 (IMC = [kg]/ [m^2^]).

**Table 1:** Anthropometric data of the sample in men and women

	Women (*N* = 598)		Men (*N* = 313)	
Age (years)	M = 21.02 years	SD = 1.99 years	M = 21.24 years	SD = 1.71 years
Weight (kgs)	M = 57.58 kgs	SD = 9.16 kgs	M = 72.83 kgs	SD = 12.35 kgs
Height (meters)	M = 1.62 m	SD = 0.70 m	M = 1.75 m	SD = 0.70 m

All participants completed the questionnaires used for the study. Yet, there were some that did not answer all the questions; thus, these were discarded. Data was recollected randomly, seeing as specific classes from each building on campus were selected. Professors were asked for a specific time span during class- either at the beginning or at the end-, for voluntary students to sign the Informed Consent letter, and answer the two instruments. Those students who were not willing to participate in the study, left the room. Six internship students from the Nutrition Clinic available on campus aided in visiting each classroom, handing out, and applying all the questionnaires. Hence, the applications were not administered directly by the researchers conducting the study.

### Instruments

The ORTO-14MX _[TSEA2]_ Questionnaire _[TSEA1]_, adapted to Mexican university students, was used to measure ON (Annex 1). The objective of the instrument is to examine obsessive behaviors in a person regarding the selection of food, its preparation and consumption habits, and attitudes towards food considered “healthy”. It is important to add that the original instrument for the Orthorexia Nervosa (ORTO-15) was previously validated in Spanish [9]. The ORTO-14MX consists of 14 questions, measured in 4 points on a Likert-type scale that goes from “always” to “never”. A score of 40 points or less means that there is a pathological behavior characterized by a strong concern for healthy eating. However, the score does not imply having a mental health disorder. The items as a group evaluate the obsessive attitude of people to choose, buy, prepare, and eat food that they consider healthy. A cut point of less than 35 has been implemented to increase specificity, and improve diagnostic trends [4, 13].

For the present research, a shorter version of the original instrument (ORTO-15) was used; its factorial structure was previously validated, precisely, in Mexican university students by Parra and her collaborators. As a result of this analysis, four factors that measure thoughts, emotions and behaviors related to food were obtained. This version contains 14 of the 15 original test items of the ORTO-15 in its original version. The overall Cronbach’s Alpha value was equal to 0.78 with 52.4% of the total variance explained. The factors [41] are:

Factor 1, called “Obsessive Cognition”, corresponds to test items 3, 4 and 7. They refer to thoughts and attitudes that generate excessive concern, and because of its duration and intensity, it’s difficult to control on a daily basis. This causes emotional discomfort and a need to stay healthy and eat “correctly.” These thoughts are hypervigilant and can be used as a coping strategy, particularly for stress.

Factor 2 was named “Rational Cognition”. It contains test items 6, 10, 11, 12 and 14. It refers to a mental and rational construction that generates logical thoughts in order to evaluate, understand and take action in what is seen as a “healthy lifestyle”. It indicates a great need to be accepted for one’s appearance, levels of self-confidence, and the desire of approval of others.

Factor 3, “Emotional Regulation”, was composed of test items 1, 2 and 13. It is a factor that measures the influence of emotions on the individual, and how he or she experiences and expresses them. In this sense, food has a strong motivational component for the individual in terms of their biological and social adaptation.

Finally, factor 4, “Normative Social”, contains test items 8, 9 and 15. It measures compliance with social rules accepted by the group they belong to and that people must follow to have a healthy coexistence with food.

In the process of validating instrument in Mexican university students, a Principal Component analysis with a Varimax rotation was run. Also, the anti-image matrix was obtained with the purpose of identifying if any item could be reducing the total explained variance. In consequence, item number 5 (For you, is the taste of food the principal criteria to determine its quality?) reduced the total explained variance from 52.5 to 49.9%.

Additionally, an analysis was run to verify if by eliminating said item, Cronbach’s Alpha decreased. There was no significative change in the Alpha value; therefore, the decision was made to only keep 14 items.

On the other hand, the detection of eating disorders was executed using the Eating Disorder Questionnaire (EDE-Q) in its short version. It was designed by Grilo, Reas, Hopwood and Crosby [42] as a measure of specific psychopathology found in eating disorders. The EDE-Q evaluates aspects such as: weight, shape, eating concerns, and food restriction. The Questionnaire uses a grading system in points: the higher the score in the questionnaire, the greater the severity of the disorder It is composed of 7 questions divided into 3 sub scales: restriction, concern for one’s shape and weight, and dissatisfaction with these two (Annex 2). They are evaluated on a scale from 0 to 6, where a higher score reflects a higher pathology symptomatology. Lastly, the Questionnaire ought to be answered considering the 28 days prior to the application; this way, frequencies are measured according to the amount of days in which disordered behaviors are present.

Despite the unusual structure of the Questionnaire, which consists of very few items that do make up factors, this version has been analyzed and validated, as well as published, and is therefore accepted as a trustworthy methodological instrument. Actually, it’s considered a viable alternative to the EDE and has shown high internal consistency [43, 44]. For example, Luce and Crowther found excellent reliability in this test [44]. Likewise, Unikel et al. [45] recently validated the instrument in Mexican women (revise Annex 1).

### Procedure

The study consisted of applying both, the EDE-Q in its short version, and the ORTO-14MX Questionnaire in its validated version for Mexican population _[TSEA3]_. The instruments were administered on a sample of university students from Mexico City. Participants were approached trhough non-probabilistic convenience, seeing as professors from indiscriminate classes were to invite their students to voluntarily participate in the study. The Questionnaires were self-administered. Those questionnaires which were not answered in their totality, were discarded from the study. For ethical reasons, the participants did not receive any financial compensation in exchange for their participation in the study.

To protect the confidentiality of all participants, they were informed that their answers would be treated anonymously and only for statistical purposes. They were asked to respond with the answer considered most appropriate to their attitude and behaviors. The Research Ethics Committee of the University approved this study, and it is in accordance with the General Health Law in México regarding research procedures. Therefore, participants signed a Consent Informed letter, included in Annex 3.

### Data analysis

The SPSS software version 25 was used to perform data analysis. Descriptive analyses were done using frequency and percentages for categorical variables, such as mean and standard deviation, for continuous measures. Furthermore, it was intended to investigate if, just like in previous studies, statistically significant differences were found between men and women regarding the total obtained scores both in the ON scale and in the Specific Symptomatology one (ED). A Student T-Test for independent simples was run for each factor that compose both scales.

To achieve the objective of this investigation, which main purpose was to analyze if the factors from the ORTO-14MX instrument managed to predict accurately and trustworthily the apparition of specific symptomatology of eating disorders, statistical analyses were run. First, a Pearson’s Correlation was used between the ORTO-14MX factors, and the total points obtained with the EDE-Q in its short version. Furthermore, a linear regression analysis by steps was applied to obtain which specific factors from the ORTO-14MX can precisely predict the probability to develop an eating disorder.

## Results

In the first place, the total scores from the scale measuring orthorexia in the sample were obtained; 61.8% of the total sample obtained scores equal or lower to 40, and 34.3% of that percentage obtained scores equal or lower to 35. Both scores are indicators of orthorexia; however, considering a cut point lower than 35 has proven higher specificity and better diagnostic tendencies. When analyzing this same percentage in women from the sample, it increases to 65.7% considering the cut point at scores equal or lower to 40, and 38.1% considering the cut point at scores equal or lower to 35. Regarding men, the initial percentage decreased to 54.7 and 27.4%, respectively. In relation to the prevalence found in Nutrition students, 75.7% scored with 40 or less in the questionnaire that measures orthorexia.

Respecting the average obtained was 12.02 for the whole sample (SD = 8.95), with a minimum score of 0 and a maximum of 42. For the sample constructed entirely of women the average calculated is 13.46 (SD = 9.32), with the same minimum and maximum scores for the whole sample. In contrast, the men’s average value is 9.36 (SD = 7.48), with a minimum of 0 and a maximum of 34 points.

Concerning the ORTO-14MX, every single factor had higher marks in men than in women. Consistently, these grades indicate higher risks of developing orthorexia in women, while a greater score from women in the EDE-Q demonstrates greater liability in specific symptomatology of ED. In relation to the comparison between men and women’s samples, statistically significant differences were found in factors 1, 2 and 3 of the scale that measures ON, as well as in every subscale from de EDE-Q; these are shown in Table 2.

**Table 2:** Descriptive statistics and differences between men and women regarding orthorexia nervosa and specific symptomatology of an ED in Mexican university students

	Women				Men	
	M	SD	M	SD	t	p
ORTO-14 MX						
Obsessive Cognition	8.78	2.33	9.35	2.10	−3.74	.00**
Rational Cognition	7.69	2.35	8.07	2.60	−2.15	.03*
Emotional Regulation	8.96	2.24	9.67	1.96	−4.91	.00**
Normative Social	7.91	1.88	8.20	1.98	−2.15	.03*
Total	35.45	6.70	37.59	6.88	−4.48	.00**
EDE-Q						
Restriction	4.50	4.39	3.56	4.00	3.24	.00**
Concern for one’s shape and weight	3.98	3.18	2.59	2.40	7.43	.00**
Dissatisfaction	4.99	3.50	3.21	3.21	7.44	.00**
Total	13.46	9.33	9.36	7.49	7.13	.00**

*P ≤ .05 **P ≤ .01

A Pearson’s correlation analysis was conducted to analyze the relationship between the subscales that make up the variable of ON, and the subscales on the EDE-Q scale in its short version. As it can be seen in Table 3, statistically significant and inversely proportional relationships were found between the three EDE-Q factors and the four factors of the ORTO-14MX. Although the correlations ranged from mild to moderate, a relationship between the presence of orthorexia and the specific symptomatology of an ED is identified. It is feasible to test the hypothesis of a possible prediction (see Table 3).

**Table 3:** Relationship between orthorexia nervosa and eating disorder specific symptomatology in university students in Mexico

	OC	RC	ER	NS	EDE-Q1	EDE-Q2	EDE-Q3
Obsessive Cognition (OC)		.450^**^	.466^**^	.287^**^	−.530^**^	−.494^**^	−.437^**^
Rational Cognition (RC)			.307^**^	.302^**^	−.335^**^	−.307^**^	−.251^**^
Emotional Regulation (ER)				.267^**^	−.429^**^	−.347^**^	−.315^**^
Normative Social (NS)					−.208^**^	−.266^**^	−.267^**^
Restriction (EDE-Q1)						.506^**^	.461^**^
Concern for one’s shape and weight (EDE-Q2)							.638^**^
Dissatisfaction (EDE-Q3)							

Note: *p ≤ .05, **p ≤ .01

### Eating disorder prediction from the factors composing the variable of orthorexia nervosa

The purpose of this paragraph is to get to know the influence that the different components of ON have over the global score obtained in the EDE-Q in university students in Mexico. Step- wise linear regression was analyzed. The results are presented in Table 4.

**Table 4:** Linear regression by steps analysis summary to predict EDE-Q total final scores from the factors that make up the ORTO-14MX scale

Step and predictive variable	B	SD B	β	R^2^	∆ R^2^
Step 1: Obsessive Cognition	−5.17	.37	−.43	.34	.349
Step 2: Emotional Dysregulation	−2.35	.37	−.19	.38	.037
Step 3: Rational Cognition	−1.38	.39	−.09	.39	.011
Step 4: Social Normative	−1.16	.43	−.08	.40	.005

**Fig. 1:**
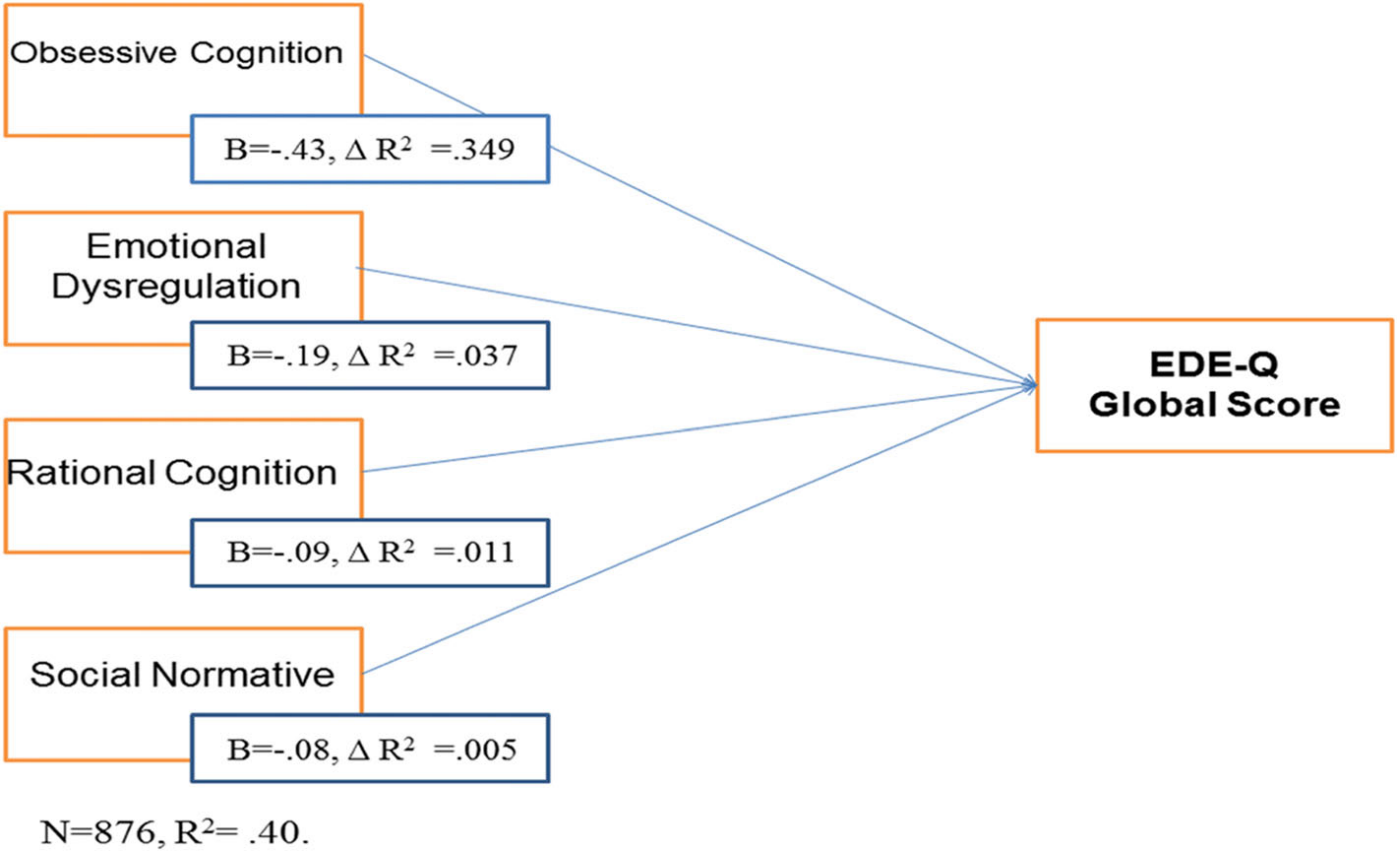
Linear regression analysis for the EDE-Q

The dependent variable used in the regression analysis corresponds to the global score from the EDE-Q in its short version. Specific symptomatology of an eating disorder was predicted in four steps in the following order. The first step involved the subscale “Obsessive Cognition” (F = 433.85, *p* = .000). The second step involved subscale “Emotional Dysregulation” (F = 249.42, *p* = .000). The third step involved factor “Rational Cognition” (F = 170.71, *p* = .000). The fourth and last step involved factor “Social Normative” (F = 133.12, *p* = .000). This can be observed in Fig. 1.

## Discussion

The present study’s main objective was to analyze if there was a possible relationship and predictive value between the factors that measure if someone has orthorexia, and the overall score from the EDE-Q, and determine if this relationship could indicate a specific’s eating disorder symptomatology amongst university students in Mexico. Additionally, the sample’s prevalence in these values was obtained, and the differences between men and women regarding these variables.

Firstly, a significant prevalence of orthorexia was found in the sample, seeing as out of the 100, 68.1% obtained scores equal or lower to 40, which is an indicator of the presence of certain pathological behavior characterized by the strong worry and desire to eat healthily. Out of this 68.1, 34.3% obtained a score equal or lower to 35. Just as it was found in the sample and its results in the present study, various authors have found higher psychosocial vulnerability in adolescent and university population, regarding eating disorder behavior. This is a critical period in which this age-group consolidates and strengthens their own behavioral and eating habits, as well as a major importance in the development of body image [22, 23]. Besides, within university students, sub-groups have been described as even in higher risk, such as students form nutrition, medicine, and sport’s science [6].

However, the authors from the present study consider it especially important to put forward two contrasting situations. On one hand, students are immersed in fast-paced lifestyles, and they lack the possibility of investing enough time and money in controlling what they eat; hence, health becomes a secondary concern. On the other, students with high obsessive cognition can be found. Due to the concern over eating “healthy”, “clean”, and “correct”, most of their personal and economic resources are invested in what is perceived that aids in keeping eating habits under control. Thus, this group of people are deprived of time and space to perform other personal, social, and integration activities because of focus on what is being consumed.

After analyzing prevalence by sex, it was found that percentages were higher in women than in men, which is consistent with what other authors have found in their own studies. It has been detected that there is a higher prevalence of eating disorders, and pathological or disordered eating in the female population. The same results could be observed for global scores obtained in the EDE-Q: women had an average score higher than men. With results in agreement with what is mentioned before, statistically significant differences were found when comparing each one of the factors that make up instruments used in women and in men. Adding on, the factors from the ORTO14-MX instrument were also statistically significant, and lower for women, whilst the factors from the EDE-Q scale in its short version showed statistically significant averages, higher in women.

Now, regarding the relationship between orthorexia nervosa and eating disorders, it can be said that researchers are still far from understanding it clearly, seeing as ON hasn’t even been included as a pathology in the DSM-5, and health professionals don’t have much information on it. This hinders even more its exploration and possible description. Actually, studies that have been published so far have precisely found that it’s those healthcare professionals who exhibit more frequently these disordered behaviors. One hypothesis as to why this happens is that more knowledge directed over healthy eating is acquired in their studies. But there’s a thin line that separates this knowledge from pathological behavior, making it quite difficult to perceive the difference. In fact, in the sample used for this study, 75.7% of students that were majoring in Nutrition obtained scores equal or lower to 40 in the instrument that measures orthorexia, which is consistent with other studies who have obtained similar prevalence values in dietitians and nutrition students. For example, Tremeling et al. found that out of 2, 500 dietitian in the United States, 49.5% were at risk of developing orthorexia, and 12% in risk of developing a diagnosed eating disorder; also noting that those at risk for ON had lower BMI values, and 8.2% self-reported having been previously in treatment for an eating disorder. These authors argued that the relationship between ON and an ED associates not only in disordered eating behaviors, but also in concern about body size and image [46].

Regarding the ability to predict an eating disorder based on the presence of orthorexia, it’s necessary to mention that a promising score was obtained, but it wasn’t conclusive, given that the obtained correlations between factors were significant with a mild to moderate magnitude, while the determination coefficient obtained from the linear multiple regression indicates that the percentage of explained variance reached a percentage of 40%, which is considered acceptable as a predictor for eating disorders when orthorexia is detected. This finding is overly valuable as a predictor (but isn’t sufficient), because this is a psychosocial study in a poorly studied Latin sample.

When the factors’ order was analyzed (obtained from the linear regression analysis), the objective was to explain specific symptomatology from eating disorders. “Obsessive cognition” was found to be the first factor in the model, also explaining most of the variance’s score, followed by “emotional dysregulation”, “rational cognition”, and “social normative” last.

Obsessive cognition is conformed by a series of persistent and involuntary thoughts, as well as high levels of concern for at least 3 hours daily over what will be eaten, through the past 3 months. Said concern is moved by the need to be physically “healthy”, regardless of concern over weight and body image. There’s also a constant search and attempt to “keep clean”, “eat clean and correctly”, and maintain control of surroundings. Along this line, diverse studies performed with patients diagnosed with different types of eating disorders, show obsessive traits in 27 to 61% of cases; shyness and dependent characteristics in 21 to 48%, and anxiety in 51 to 64% of cases. Earlier research on this topic agrees that the prevailing personality type to develop an eating disorder combines obsessive, inhibition, and conformism traits [47]. Therefore, there is a relationship between thought rumination, and obsessive personality traits.

The second factor, “emotional dysregulation”, is composed by the presence of confusion, anxiety and preoccupation, and the difficulty to stay calm in situations where the person doesn’t have control partially or wholly. Congruently, the Minnesota Multiphasic Personality Inventory (MMPI) has provided relevant information about general characteristics found in affective states (sadness, pessimism, euphoria, etc.), the quality of interpersonal relationships (difficulty in establishing deep affective compromise), and excessive demanding and expressing characteristics of affection in patients with an eating disorder (or at risk of developing one). Lastly, people with an ED usually have trouble with impulse control and tolerating frustration. As a matter of fact, it is esteemed that around 53 to 93% of patients with an eating disorder have personality alterations [48]. Likewise, the simultaneous manifestation of more than one personality disorder in said samples, especially in patients diagnosed with bulimia nervosa and previous story of anorexia nervosa, has been documented [49, 50].

In what refers to the “rational cognition” and “social normative” factor, it can be said that despite their inclusion in the model, the variance’s percentage explained through them is quite low. In this case, it is hypothesized that the presence of both obeys socioeconomic and cultural characteristics, rather than individual ones. These 2 factors include items related to spending more money to eat more healthily, or the perception of types of foods sold at local markets and supermarkets. About this, it’s relevant to explain that in Mexico “healthy” or “wholesome” food is considerably more expensive than fast food or “garnachas”, which are also more easily found to buy. Additionally, Mexican population consists of a collectivist culture. This means that it’s almost compulsory to eat accompanied by other members of endogroups, or interact in every context associated with food, despite individual personality traits [51, 52].

In conclusion, the cultural factor permeates every psychosocial behavior, such as eating. Although eating is an act of survival biologically determined, it is also immersed in society. In this sense, eating behaviors are profoundly shaped by the person’s context, involving cultural, social, and environmental contexts [35]. The interaction found between sociocultural context and ON has been scarcely studied in Mexican population; most studies of this type have been made with European, Turkish, and American samples [36].

Ergo, it’s necessary to consider the importance given to food as an interaction ritual in Mexico, besides being used as a tool to unite people. Mexican gastronomy is loaded with collective emotions because of the country’s history and endoculturation found inside families. Consequently, it’s not a simple task to compare the findings of this study with those obtained in nations considered individualistic. Plus, Mexican people have rather high levels of social desirability. In turn, this becomes a potential source of internal invalidation in every research that uses instruments such as interviews and self-reports [53].

## Conclusions

In conclusion, the objectives of this investigation were achieved. This study analyzes the existing relationship between the composing factors of the ORTO-14MX and the factors from the Eating Disorders Questionnaire (EDE-Q) in its short version. It was later found that these factors that indicate orthorexia nervosa (ON) accurately predict to some degree eating disorders in university students from Mexico City. Additionally, it analyzed statistical differences found between men and women, according to these same variables.

The factors from the ORTO-14MX and the EDE-Q don’t have a 100% predictive capacity. However, it does have a significant enough capacity to be said that it is very valuable as an initial screening instrument that manages to give quick and reliable information on the mental state of the person regarding eating habits (which is a very complex phenomena). If the fact that most of the habits acquired during adolescent and early-adulthood years is taken in consideration, this period is of utmost importance and gives a worthwhile opportunity to evaluate predictive factors, and risk factors to develop eating disorders.

Given that there are not enough elements to stablish a definite cut-point, a score equal to 32 based on the value that marks the superior limit in the first quarter 32 ≥ is tentatively proposed. However, this value will be corroborated or adjusted according to other researchers and their usage of the ORTO14-MX in clinical and non-clinical samples.

It is necessary to consider taking into account these traits from orthorexia grave and important enough to offer integral treatment since this moment, and not wait until a full-on eating disorder has developed to offer help or even seek for help. For this to happen, there must be more information accessible for sanitary professions in general: Nutrition is one of the most in need, but also students and practitioners from Medicine, Nursing, Social Work, and Psychology, amongst others. The more interdisciplinary the knowledge, the more tailored evaluation and intervention strategies can be. Regarding the differences in the frequency of ON found among the Italian, Polish and Spanish samples, we cannot exclude that a role is played by gastronomic culture, Mediterranean diet, convivial and social value attributed to eating and main approaches aimed at improving one’s health typical of each socio-cultural context. Disordered eating behavior rarely has to do completely with losing weight or being healthy; it’s a priority for researchers in this field to study and integrate the relevance of society and different systems in how a person views eating, and the purpose of their eating habits. That is, after all, what makes the study of eating disorders so complex and arduous: family, friends, school, workplaces, and general socio-economic markets will inevitably influence how food, health, and the body is viewed.

### Limitations and suggestions

The results obtained in this research highlight the long path that is still to be travelled by the scientific community, for orthorexia to be considered a new eating disorder that can be included in the diagnostic manuals. The relationship between eating disorders and orthorexia is, undoubtedly, intricate, and is far from being understood. Additional studies are needed to describe specific behaviors from people with orthorexia (its etiology, diagnosis, treatment, and prevention). In order to convincingly affirm predictions between orthorexia and specific symptomatology of eating disorders, comparisons must be made with clinical samples. The sample used in this subject was taken from university students, and it was not a heterogeneous nor generalizable sample. However, using a non-clinical sample can help generalize results to more people, therefore being a possible strength. Also, other confirming instruments, rather than screening or self-report instruments, could be used to confirm a presumed diagnostic, both for ON and for ED. Likewise, this study was only applied in a specific socioeconomic background. More studies comparing scores from other backgrounds would be quite useful, particularly done with low social classes, students from public universities, and students from different geographical regions of the country.

## Data Availability

The data sets used and analyzed during the current study are available from the corresponding author on reasonable request.

## Abbreviations

ON: Orthorexia Nervosa
AN: Anorexia Nervosa
ED: Eating Disorder
DSM-5: Diagnostic and Statistical Manual of Mental Disorders
ICD-10: International Classification of Diseases
BOT: Bratman’s Orthorexia Test
EDE: Eating Disorder Examination
EDE-Q: Eating Disorder Examination Questionnaire
